# Percutaneous Coronary Intervention to Spontaneous Coronary Artery Dissection With Coexistent Takotsubo Cardiomyopathy: A Case Report

**DOI:** 10.1002/ccr3.72462

**Published:** 2026-04-05

**Authors:** Ahmed Elsherif, Pouya Ebrahimi, Sophia Khattak, Sudhakar George, Sohail Q. Khan

**Affiliations:** ^1^ Department of Cardiology Queen Elizabeth Hospital, University Hospitals Birmingham NHS Foundation Trust Birmingham UK; ^2^ Institute of Cardiovascular Sciences, University of Birmingham Birmingham UK

**Keywords:** acute coronary syndrome, percutaneous coronary intervention, spontaneous coronary artery dissection, takotsubo cardiomyopathy

## Abstract

Spontaneous coronary artery dissection (SCAD) is an uncommon cause of non‐atherosclerotic acute coronary syndrome (ACS) resulting in myocardial ischemia and ventricular wall motion abnormalities (WMA). SCAD and Takotsubo Cardiomyopathy (TC) share a common risk factor profile and clinical characteristics. We report a 55‐year‐old female patient who presented with chest pain and elevated troponin level. Coronary angiography (CA) showed tortuosity in the first diagonal (D1) artery suspicious of SCAD. Left ventriculogram showed typical apical ballooning of TC. Because of ongoing chest pain during the procedure, we proceeded to percutaneous coronary intervention (PCI) to D1. Intracoronary optical frequency domain imaging (OFDI) of D1 showed intramural hematoma extending to proximal LAD and treated with a 2.25 × 12 mm Promus Element drug‐eluting stent (DES). Transthoracic Echocardiography (TTE) showed mid anterolateral, inferolateral, and all apical hypokinesia of LV with preserved basal segments and mildly impaired systolic function. TTE after 3 months showed fully recovered LV systolic function with resolution of WMA. Repeat CA 6 months later demonstrated healing of SCAD and OFDI showed resolution of the intramural hematoma, and cardiac MRI showed normal LV systolic and full‐thickness delayed enhancement in only the lateral wall.

## Key Clinical Message

- Spontaneous coronary artery dissection (SCAD) and takotsubo cardiomyopathy (TC) are emerging causes of myocardial infarction with non‐obstructive coronary arteries (MINOCA), and their coexistence has been reported with several shared common manifestations. While conservative management is standard, selected patients with persistent symptoms may benefit from coronary revascularisation.

## Graphical abstract

A case of spontaneous coronary artery dissection (SCAD) of the first diagonal branch (D1) with co‐existent Takotsubo Cardiomyopathy (TC), successfully revascularised with percutaneous coronary intervention (PCI).

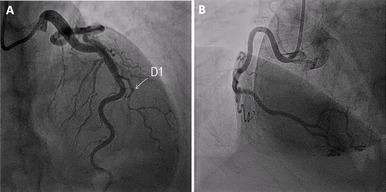

**Abbreviations**
ACS: Acute coronary syndrome
CA: Coronary Angiography
LVgram: left ventriculogram
MINOCA: Myocardial Infarction with Normal Coronary Arteries
OFDI: intracoronary optical frequency domain imaging
PCI: Percutaneous Coronary Intervention
SCAD: Spontaneous Coronary Artery Dissection
TC: Takotsubo Cardiomyopathy
TEE: Transthoracic Echocardiography
WMA: Wall Motion Abnormalit

## 1 Introduction

Takotsubo cardiomyopathy (TC), also known as stress‐induced cardiomyopathy, is a distinctive cause of reversible cardiomyopathy more commonly seen in postmenopausal females, and its clinical presentation is indistinguishable from acute coronary syndrome (ACS). Electrocardiogram (ECG) changes consistent with transient ST‐segment elevation and T‐wave inversion may present along with a variable rise in cardiac troponin level mimicking ACS [1]. Patients may show a pattern of hypo/akinesia of the mid and apical segments of the left ventricle (LV) with sparing of basal segments, resulting in the characteristic apical ballooning of TC on left ventriculogram (LVgram) [2]. In many cases, TC was preceded by stressful physical or emotional triggers, and due to its association with stress, the role of catecholamine surge in its pathogenesis has been under consideration [3].

Spontaneous coronary artery dissection (SCAD) is an epicardial coronary artery dissection and is a leading cause of non‐atherosclerotic ACS affecting young healthy women [4]. The principal mechanism of myocardial injury in SCAD is obstruction of a coronary artery due to the formation of an intramural hematoma or intimal disruption as opposed to rupture of atherosclerotic plaque or intraluminal thrombi seen in atherosclerotic coronary artery disease (CAD) [5].

Coexistence of SCAD and TC has been reported previously [6, 7]. Although conservative medical therapy has always been recommended in the management of both conditions, we present a unique case where coronary revascularization of SCAD with coexisting TC relieved the ongoing severe chest pain, followed by recovery of left ventricular (LV) systolic function and ventricular wall motion abnormalities (WMA) with a timeline of events (Table 1).

**TABLE 1:** Timeline.

Timeline	Events
Day 1	Patient presented with crushing chest pain. Electrocardiogram revealed Right Bundle Branch Block (RBBB) morphology along with elevated high‐sensitive Troponin‐I (hs‐cTnI) to 2588 ng/L. She was taken for emergent coronary angiography (CA). First diagonal artery (D1) showed luminal irregularities suspicious for spontaneous coronary artery dissection (SCAD). Left ventriculogram (LV gram) was done and showed mid and apical hypokinesia with sparing of basal segment and characteristic systolic apical ballooning of takotsubo cardiomyopathy (TC). Because of ongoing chest pain during procedure, percutaneous coronary revascularization (PCI) to D1 was performed. Optical Frequency Domain Imaging (OFDI) in D1 confirmed the diagnosed and showed hematoma extending to the proximal LAD. Transthoracic Echocardiography (TTE) revealed mid anterolateral, inferolateral and all apical segments hypokinesia with preserved basal wall motion and mildly impaired LV systolic function, ejection fraction (EF) ~45%.
After 3 months	*Repeat TTE after 3 months showed complete* resolution of the left ventricular wall motion abnormalities (WMA), with *full regained LV systolic function* (EF ~60%).
After 6 months	Cardiac MRI was performed after 6 months and demonstrated normal LV systolic function with full‐thickness delayed enhancement in only 2 segments of the lateral wall, corresponding to the D1 territory.

## 2 Case History/Examination

**FIGURE 1:**
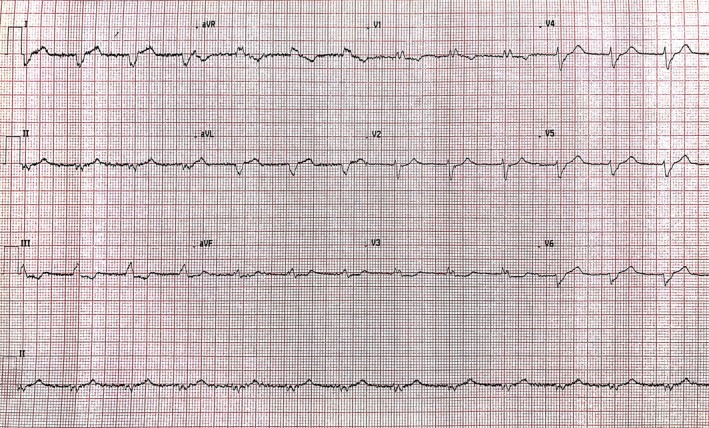
Initial electrocardiogram (ECG) at presentation showed Right Bundle Branch Block (RBBB) morphology.

A 55‐year‐old woman presented to the Emergency Department (ED) with chest pain following a stressful day as a carer during which she had to perform multiple lifting and hoisting procedures. She was reassured and discharged home. She presented again a week later with worsening substernal chest pain radiating to the left shoulder for 6 h prior to her presentation. She has a background history of polycystic kidney disease (PKD) with preserved renal function and stable IgG multiple myeloma disease after treatment with VTD chemotherapy. She was hemodynamically stable with blood pressure 110/80 mmHg and HR 82 bpm, and physical examination was unremarkable. Electrocardiogram (ECG) on presentation showed right bundle branch block (RBBB) morphology (Figure 1). Routine laboratory tests revealed: creatinine 88 μmol/L and eGFR 72 mL/min/1.73 m, hemoglobin 117 g/L, platelets 175 × 10^9^/L. Cardiac biomarkers showed peak high‐sensitivity troponin T of 456 ng/L (normal < 14 ng/L). NT‐proBNP levels were not measured during the acute presentation.

## 3 Differential Diagnosis, Investigations and Treatment

**FIGURE 2:**
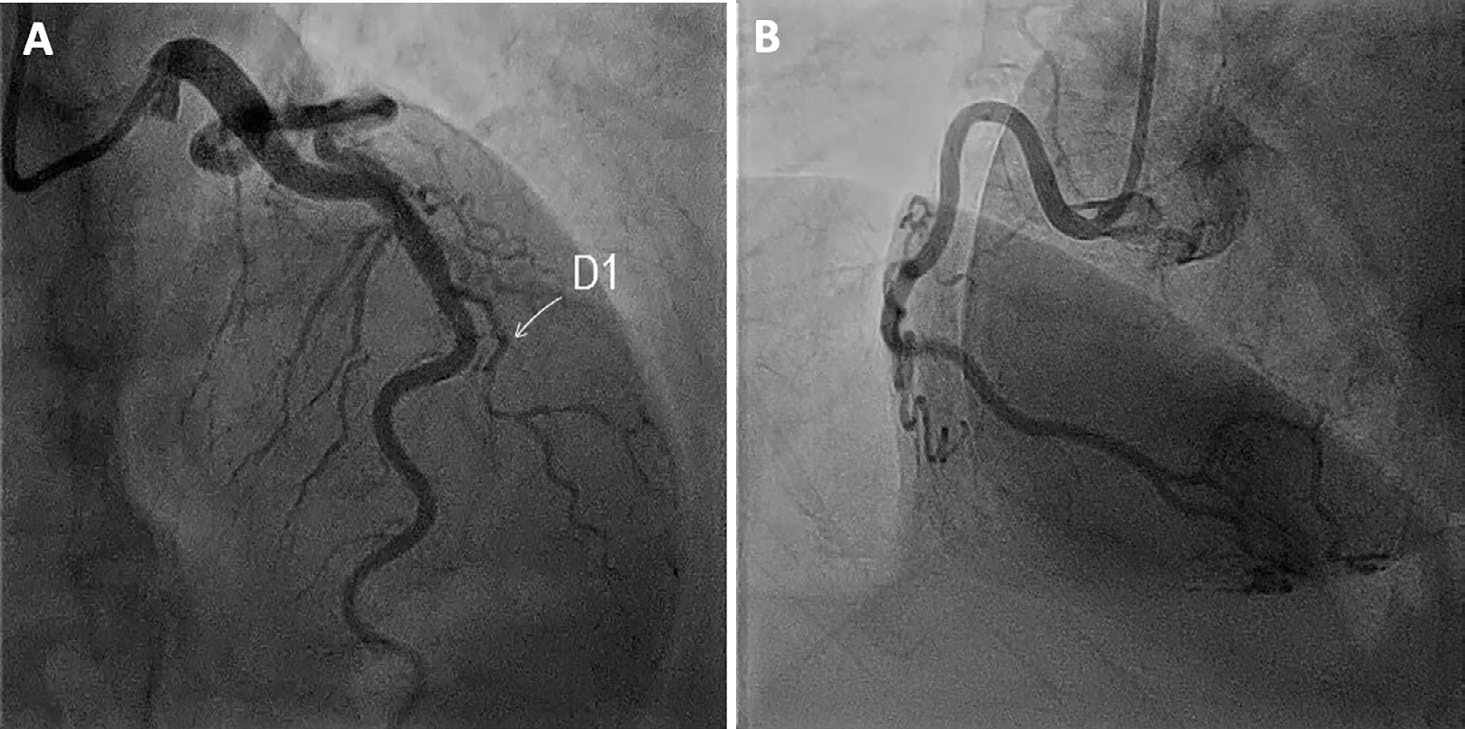
Initial coronary angiogram. (A) Normal left anterior descending artery (LAD) and a small first diagonal branch (D1) with luminal irregularities (white arrow). (B) Dominant and normal right coronary artery (RCA).

**FIGURE 3:**
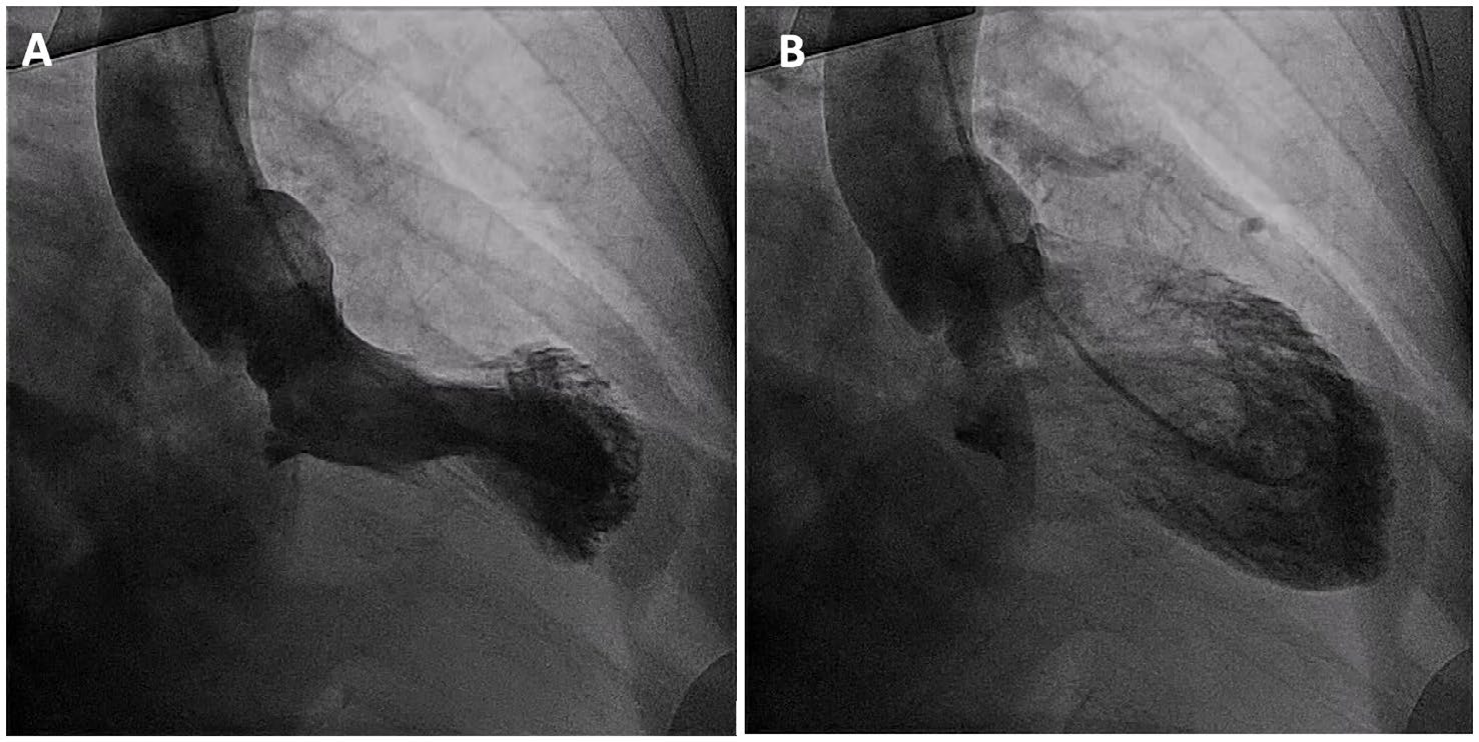
Left ventriculogram in RAO view during (A) systole and (B) diastole showing characteristic systolic apical ballooning and hyperdynamic basal segments, consistent with Takotsubo cardiomyopathy (TC).

Patient continued to experience severe unremitting chest pain despite medical therapy, including IV opioids and glyceryl trinitrate (GTN). Therefore, she was given a loading dose of aspirin 300 mg and prasugrel 60 mg and urgently transferred to the cardiac catheterization lab for emergent coronary angiography (CA). The procedure demonstrated normal coronary arteries and luminal irregularities in the first diagonal branch (D1) of the left anterior descending (LAD) artery (Figure 2) (Video 1). Left ventriculogram was performed and revealed mid and apical severe hypokinesia with apical ballooning and hyperdynamic basal segments, consistent with the diagnosis of TC (Figure 3) (Video 2). Given the emerging data on the association of TC with SCAD, this increased our suspicion for a possible SCAD in D1.

Although guidelines suggest conservative treatment of SCAD, we exceptionally decided to proceed with percutaneous coronary intervention (PCI) to D1 because of persistent severe chest pain not relieved by analgesics and intracoronary nitrates and associated with ischemic ECG changes during the procedure. This was undertaken after discussion with the patients regarding the risks and benefits. All epicardial coronary vessels were carefully assessed during the index angiography. The left main stem, proximal and mid LAD, left circumflex and its branches, and the entire right coronary artery showed normal angiographic appearance without any features suggestive of dissection (such as radiolucent intimal flaps, contrast staining, or luminal irregularities).

**FIGURE 4:**
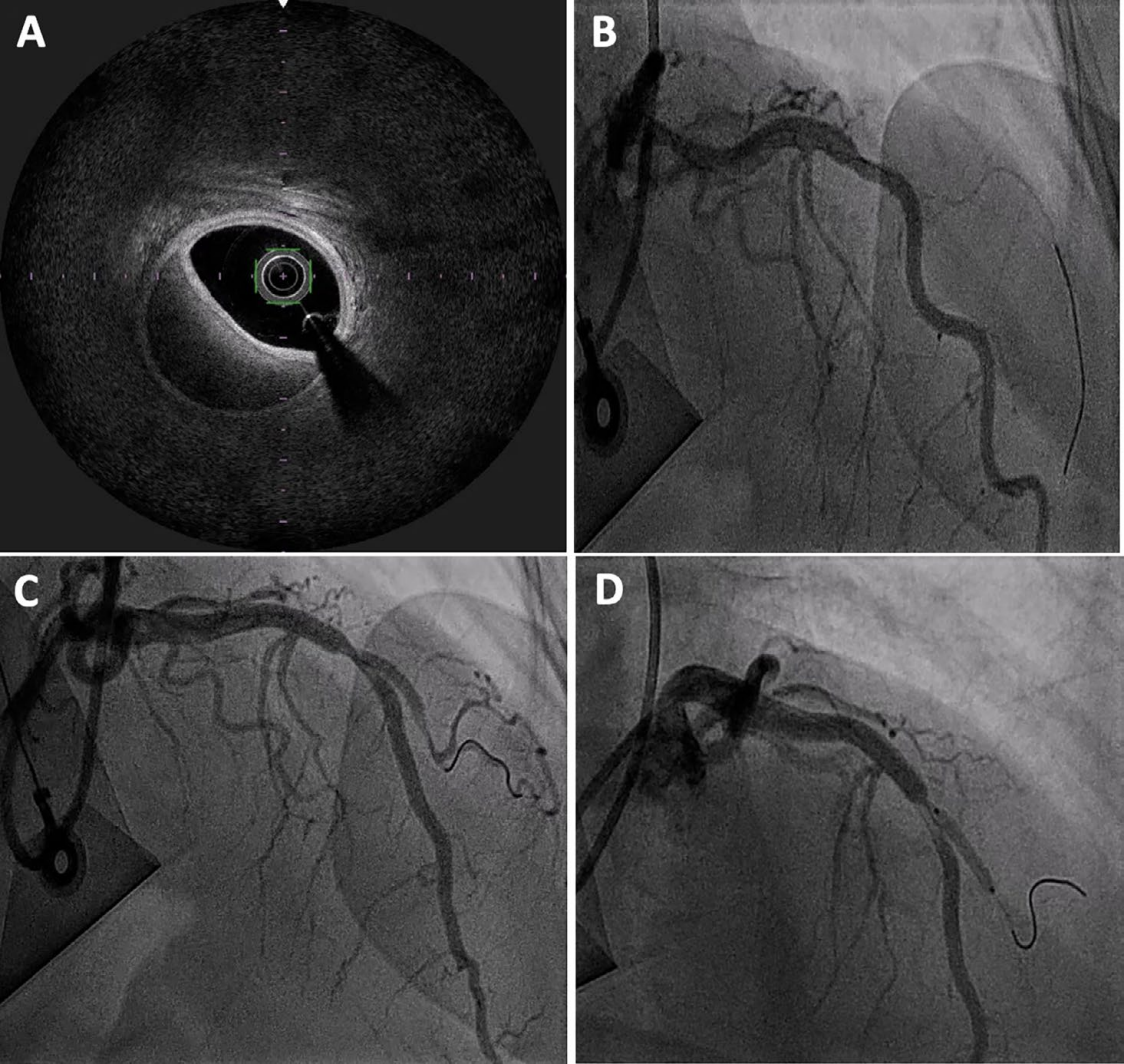
Percutaneous Coronary Intervention (PCI) to D1. (A,B) Intracoronary optical frequency domain imaging (OFDI) of LAD showed intramural hematoma and confirmed wire position into true lumen. (C) Predilatation of the diagonal branch was performed using a 2.0 × 15 mm semi‐compliant balloon. (D) A 2.25 × 12 mm Promus drug‐eluting stent (DES) was then deployed.

We advanced a runthrough wire to the distal LAD, and a second runthrough wire was placed into the D1. Intracoronary optical frequency domain imaging (OFDI) OFDI of the D1 was performed using a Dragonfly imaging catheter (Abbott Vascular), which requires clearance of intravascular blood by injecting contrast during the image recording. This must be carried out carefully because contrast injection can propagate the coronary dissection. The OFDI confirmed the presence of intramural hematoma extending into the proximal LAD but demonstrated that the guidewire was positioned in the true lumen. Intracoronary imaging of other non‐culprit vessels was not performed during the index procedure due to their normal angiographic appearance. Pre‐dilatation was performed cautiously with a 2.0 × 15 mm semi‐compliant balloon at nominal pressure to avoid excessive force that could propagate the dissection. A 2.25 × 12 mm Promus Element drug‐eluting stent (DES) was then deployed at 12 atm, with careful attention to avoid geographic miss or edge dissection, achieving TIMI III flow (Figure 4).

**FIGURE 5:**
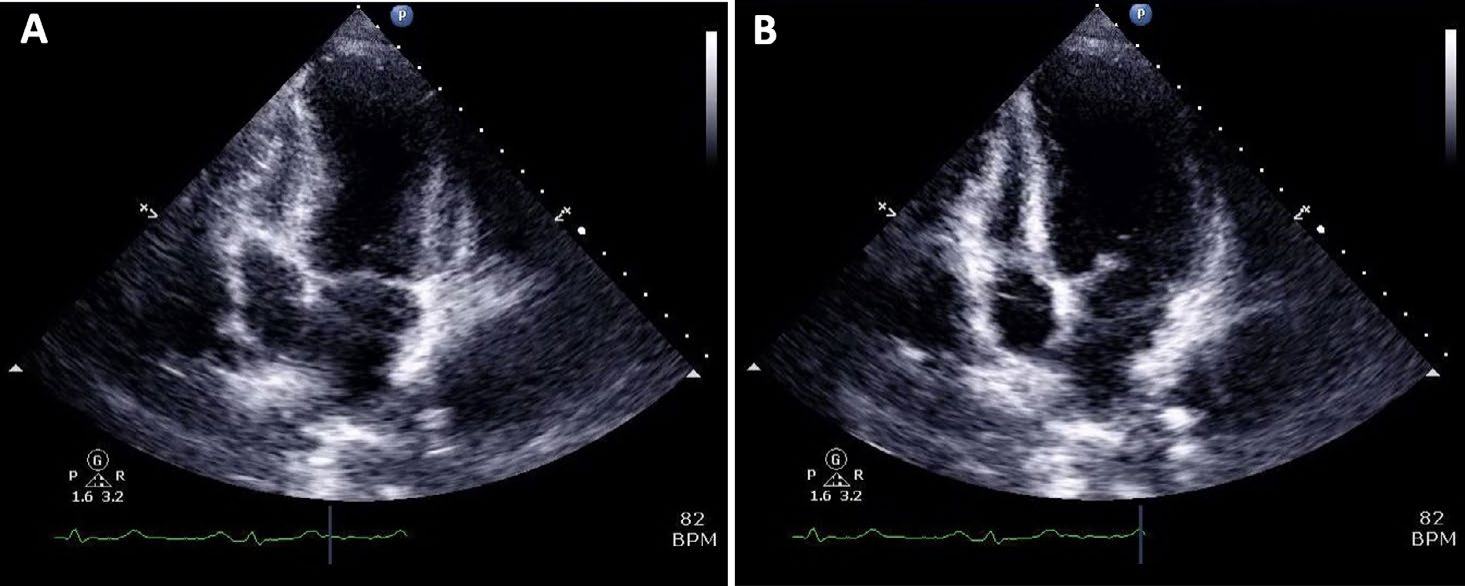
Transthoracic echocardiography (TTE), A4C view during (A) systole and (B) diastole. They revealed mid anterolateral, inferolateral, and all apical segments hypokinesia with preserved basal wall motion, consistent with the diagnosis of TC.

Transthoracic Echocardiography (TTE) was performed on the day of the procedure revealed mid anterolateral, inferolateral, and all apical segments showing hypokinesia with preserved basal wall motion in all segments, and left ventricular ejection fraction (LVEF) was mildly impaired at 45% (Figure 5). Cardiac MRI was not performed during the acute phase of presentation, which represents a missed opportunity to demonstrate myocardial edema using T2‐weighted or T2‐mapping sequences.

The patient remained hemodynamically stable with no chest pain after PCI. She was discharged the following day on medical treatment with Aspirin 75 mg once a day, Prasugrel 10 mg once a day, Bisoprolol 2.5 mg once a day, Ramipril 2.5 mg once a day, and Atorvastatin 40 mg once a day.

## 4 Outcome and Follow‐Up

Follow‐up TTE at 3 months showed complete resolution of the LV wall motion abnormalities in all previously affected segments, with full recovery of *LV systolic function to* EF ~60%. Notably, all segments beyond the D1 territory that were initially dysfunctional demonstrated complete normalization, consistent with myocardial stunning rather than permanent ischemic injury. An ultrasound of the femoral and carotid arteries did not show any evidence of fibromuscular dysplasia.

**FIGURE 6:**
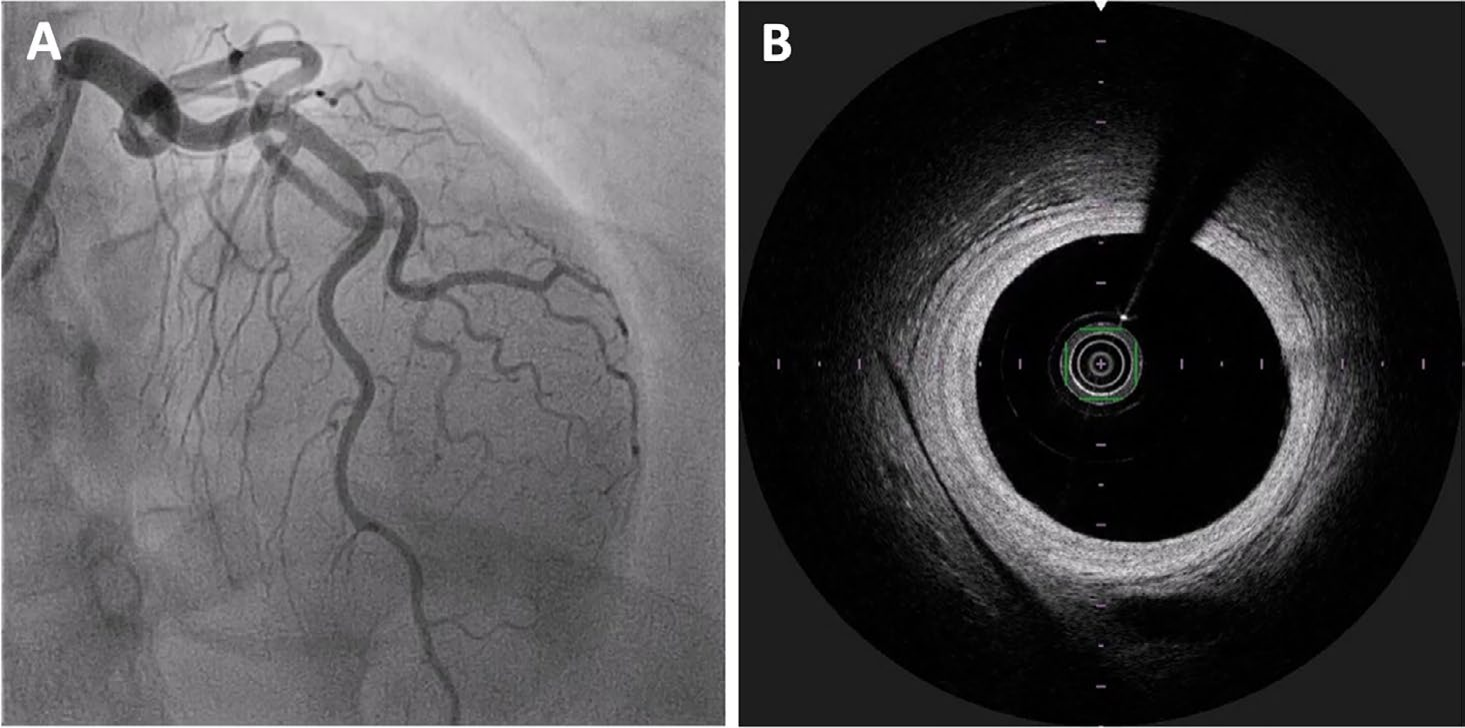
Follow‐up coronary angiography at 6 months. (A) It demonstrated angiographic healing of the SCAD of D1 with an increase in vessel caliber. (B) Repeat OFDI confirmed complete resolution of the intramural hematoma within the D1.

At 6 months follow‐up, we repeated the CA with OFDI to D1 to assess the stent and ensure healing of the intramural hematoma. The CA showed angiographic healing of the SCAD of D1 with an increase in vessel caliber (Video 3), and OFDI showed complete resolution of the hematoma with optimum stent expansion and opposition (Figure 6).

A cardiac MRI was also performed after 6 months and demonstrated normal LV systolic function with full‐thickness delayed enhancement in only 2 segments of the lateral wall, corresponding to the D1 territory, while all other previously dysfunctional segments showed no evidence of infarction. This selective pattern of scar formation with complete functional recovery of all other segments strongly supports the diagnosis of coexistent SCAD and transient TC.

**VIDEO 1:** Initial coronary angiogram showed normal left anterior descending (LAD) artery and a small first diagonal branch (D1) with luminal irregularities. Video content can be viewed at https://onlinelibrary.wiley.com/doi/10.1002/ccr3.72462.

**VIDEO 2:** Left ventriculogram in RAO view showing systolic apical ballooning due to hypokinesis and spared basal, consistent with Takotsubo cardiomyopathy. Video content can be viewed at https://onlinelibrary.wiley.com/doi/10.1002/ccr3.72462.

**VIDEO 3:** Follow‐up coronary angiography at 6 months demonstrated angiographic healing of the SCAD of D1 with an increase in vessel caliber. Video content can be viewed at https://onlinelibrary.wiley.com/doi/10.1002/ccr3.72462.

## 5 Discussion

This case illustrates a middle‐aged woman without pre‐existing atherosclerotic risk factors presenting with symptoms consistent with ACS, coronary angiographic appearance suspicious of SCAD to D1, and presumed TC by a systolic apical ballooning on ventriculogram. Although SCAD and TC are mostly two distinct non‐atherosclerotic causes of ACS, there are reported cases in the literature showing coexistence. There is, however, an absence of guidelines or expert consensus on the benefits of coronary revascularization of SCAD on improving the LV myocardial function in the context of coexistent TC [6]. The diagnosis of coexistent SCAD and TC in our patient is supported by the pattern of regional wall motion abnormalities observed on echocardiography. The first diagonal branch typically supplies the anterolateral wall of the left ventricle, whereas in our patient, the wall motion abnormalities extended beyond the D1 territory to include mid inferolateral segments (left circumflex artery territory) and all four apical segments. This extensive pattern of dysfunction, with characteristic apical ballooning beyond the distribution of the LAD, could not be explained by ischemia from the isolated D1 SCAD alone. Additionally, OFDI confirmed that the LAD itself was patent despite the intramural hematoma extension, making it unlikely that LAD ischemia contributed to the extensive apical dysfunction. We acknowledge several limitations in our diagnostic workup. Cardiac MRI was not performed during the acute phase, which would have been ideal to demonstrate myocardial edema in the non‐infarct segments using T2‐weighted sequences. Natriuretic peptide levels (BNP or NT‐proBNP) were not measured during the acute presentation. A disproportionate elevation of natriuretic peptides relative to troponin would have provided additional supportive evidence for TC.

Although most ACS cases are due to atherosclerotic conditions, TC and SCAD are emerging causes of myocardial infarction with non‐obstructive coronary arteries (MINOCA) sharing several common features. Females are more commonly affected, and in both, the presentation can be variably associated with typical clinical manifestations of ACS, arrhythmias, or sudden cardiac death. The clinical overlap of SCAD and TC represents a complex diagnostic and therapeutic challenge. The pathophysiology link between these conditions remains incompletely understood. Recent studies suggest shared mechanisms including reversible microvascular dysfunction secondary to adrenergic surge during emotional or physical stress may act as a trigger for the development of both SCAD and TC [7].

Although a wrapped LAD SCAD can give apical ballooning, a retrospective study found that LAD SCD was present in only 2.5% of patients who developed co‐existent TC. In addition, there have been several SCAD cases reported with the unique characteristic LV regional wall motion abnormalities of TC [8, 9]. Acute mechano‐cardiac coronary artery disruption (AMCAD) term has been proposed, which suggests that SCAD is a secondary coronary event instigated by the TC. It explains that the external torsional force induced by the segmental wall motion abnormalities associated with TC results in a hinge‐point distortion of coronary arteries and results in SCAD [10].

A case series consisting of 327 SCAD patients showed precipitating emotional stress factors in 48.3%, physical stress factors in 28.1%, and connective tissue disorders such as Ehlers Danlos syndrome and Marfan syndrome in 4.9% of cases [11]. There is an obvious overlap between these two conditions in their clinical presentations; however, the main difference is that segmental regional wall motion abnormalities in TC extend beyond a single epicardial distribution as opposed to the focal territorial abnormalities in SCAD [12]. The potential contribution of the underlying medical conditions to the development of SCAD and TC warrants consideration. Patient's myeloma was in complete remission with no evidence of active disease at the time of presentation, and she had preserved renal function with her polycystic kidney disease. Therefore, the SCAD‐TC event in our patient was likely related to the well‐established triggers shared by both conditions (stress) rather than her underlying medical comorbidities.

In 52% of SCAD cases the left anterior descending (LAD) artery is affected, resulting in apical ventricular wall abnormalities that are identical to those observed in TC [13]. Interestingly, in our case the LAD was patent. Intracoronary imaging with optical frequency domain imaging (OFDI) helped us to confirm SCAD after revascularization of D1 by seeing proximal extension of intramural hematoma to the LAD. It may therefore be prudent to investigate for SCAD in patients presenting with ACS symptoms, non‐obstructive coronaries, and evidence of TC on left ventriculogram.

Evidence‐based treatment strategies, specifically for patients with coexistent SCAD and TC, remain absent, necessitating individualized approaches based on clinical judgment.

The diagnosis and management of SCAD should be guided by the international expert consensus, which emphasizes multimodality imaging and conservative management given the high rates of procedural complications and evidence that most SCAD heal spontaneously over weeks to months. The decision to perform PCI in SCAD patients remains controversial and must be carefully individualized [5, 14].

Conservative therapeutic treatment of SCAD patients provided excellent long‐term outcomes. A prospective two‐year follow‐up study of 45 SCAD patients managed with a conservative strategy demonstrated complete resolution of the dissection in 54% without any incidental myocardial infarction or sudden cardiac death [15]. There is a lack of sufficient data supporting the role of PCI in *SCAD patients. Sharma* et al. *(2019) demonstrate the safe and effective use of a large 4 mm cutting balloon to treat an RCA SCAD, resulting in complete resolution of hematoma* [16].

*There are ongoing randomized trials, including the β‐Tako trial evaluating beta‐blocker therapy in TC and the BA‐SCAD trial assessing the role of beta‐blockers and antiplatelets therapy in SCAD*, which *may provide future insights into the optimal management of patients with SCAD‐TC overlap* [17, 18].

To our best knowledge, we present a unique case of PCI to a SCAD and coexistent TC, where a repeat coronary angiogram after 6 months demonstrated healed D1 with completely recovered LV systolic function. Specific trials addressing the management of SCAD‐TC overlap are lacking. Further research is needed to establish evidence‐based treatment algorithms for this complex clinical presentation.

In conclusion, there is emerging data showing an association between SCAD and TC in clinical practice. Although conservative management is preferred in both conditions, the role of coronary revascularization of SCAD with coexistent TC remains undefined. In selected cases with ongoing chest pain and persistent ischemia, as demonstrated in our case, coronary revascularization may be required to relieve the symptoms. However, prospective studies are needed to *determine the clinical relevance* of this association and the role of coronary revascularization.

## Author Contributions

**Ahmed Elsherif:** writing – original draft. **Pouya Ebrahimi:** writing – review and editing. **Sophia Khattak:** writing – review and editing. **Sudhakar George:** supervision. **Sohail Q. Khan:** writing – review and editing.

## Funding

The authors have nothing to report.

## Disclosure

Third‐Party Involvement: No persons or third‐party services were involved in the research, data analysis, or manuscript preparation who are not listed as authors. The study was entirely conceptualized, conducted, and written by the listed authors.

## Ethics Statement

This study is a single‐patient case report based on a retrospective review of the patient's clinical course. Formal ethical approval was not required because the study did not involve any experimental intervention beyond standard clinical care.

## Consent

Written informed consent was obtained from the patient for publication of this case report and any accompanying images.

## Data Availability

Data sharing is not applicable to this article as no datasets were generated or analyzed during the preparation of this case report.
